# Current Progress of CAR-NK Therapy in Cancer Treatment

**DOI:** 10.3390/cancers14174318

**Published:** 2022-09-02

**Authors:** Zhaojun Pang, Zhongyi Wang, Fengqi Li, Chunjing Feng, Xin Mu

**Affiliations:** 1 School of Pharmaceutical Science and Technology, Tianjin University, Tianjin 300072, China; 2 Tianjin University and Health-Biotech United Group Joint Laboratory of Innovative Drug Development and Translational Medicine, Tianjin University, Tianjin 300072, China; 3 Health-Biotech Group Stem Cell Research Institute, Tianjin 301799, China

**Keywords:** CAR-T, CAR-NK, immunotherapy, cancer, tumor

## Abstract

**Simple Summary:**

Chimeric antigen receptor (CAR)-T and -natural killer (NK) therapies are promising in cancer treatment. CAR-NK therapy gains great attention due to the lack of adverse effects observed in CAR-T therapies and to the NK cells’ unique mechanisms of recognizing target cells. Off-the-shelf products are in urgent need, not only for good yields, but also for lower cost and shorter preparation time. The current progress of CAR-NK therapy is discussed.

**Abstract:**

CD8^+^ T cells and natural killer (NK) cells eliminate target cells through the release of lytic granules and Fas ligand (FasL)-induced target cell apoptosis. The introduction of chimeric antigen receptor (CAR) makes these two types of cells selective and effective in killing cancer cells. The success of CAR-T therapy in the treatment of acute lymphoblastic leukemia (ALL) and other types of blood cancers proved that the immunotherapy is an effective approach in fighting against cancers, yet adverse effects, such as graft versus host disease (GvHD) and cytokine release syndrome (CRS), cannot be ignored for the CAR-T therapy. CAR-NK therapy, then, has its advantage in lacking these adverse effects and works as effective as CAR-T in terms of killing. Despite these, NK cells are known to be hard to transduce, expand in vitro, and sustain shorter in vivo comparing to infiltrated T cells. Moreover, CAR-NK therapy faces challenges as CAR-T therapy does, e.g., the time, the cost, and the potential biohazard due to the use of animal-derived products. Thus, enormous efforts are needed to develop safe, effective, and large-scalable protocols for obtaining CAR-NK cells. Here, we reviewed current progress of CAR-NK therapy, including its biological properties, CAR compositions, preparation of CAR-NK cells, and clinical progresses. We also discussed safety issues raised from genetic engineering. We hope this review is instructive to the research community and a broad range of readers.

## 1. T-Cell Therapy in Cancer Treatment

The cells used in T-cell therapy are CD8^+^ T lymphocytes, whose activated forms bear the ability to kill target cells and are thus also known as cytotoxic T lymphocytes (CTLs). To be more specific, they are αβ T cell receptor (TCR)-encoding CD8^+^ T cells [1]. It is noteworthy that there is a group of CD8^+^ T lymphocytes that express γδ TCRs, and these cells recognize target cells in an HLA-independent manner. They have distinct biological features that are not discussed here. Excellent reviews on γδ T cells can be found elsewhere [2,3]. Experiments have shown that CTLs have the ability to kill tumors. For example, autologous CTLs expressing murine TCR targeting the human carcinoembryonic antigen were used to treat three patients with metastatic colorectal cancer who had not responded to standard therapy, with one patient having an objective regression of their metastatic cancer that had spread to the lung and liver [4]. However, studies in mice and humans have shown that naturally occurring CTLs are inefficient in restricting or killing cancer cells. On the one hand, over 90% of infiltrated CTLs fail to sense tumor targets, as many of them are viruses that recognize CTLs [5]. On the other hand, tumor cells can survive these infiltrated effector cells via the tumor microenvironment (TME), which has the features of hypoxia, nutrient limitations, and abnormal vasculature and is enriched with immunosuppressive cells such as myeloid-derived suppressor cells (MDSCs), regulatory T (Treg) cells, tumor-associated macrophages (TAMs), and immunosuppressive cytokines, such as interleukin-10 (IL-10), IL-35, and transforming growth factor beta (TGF-β) [6]. Therefore, it is critical to clinically enhance the targeting of immune effector cells and to make them easier to activate upon the recognition of cancer cells.

**Figure 1:**
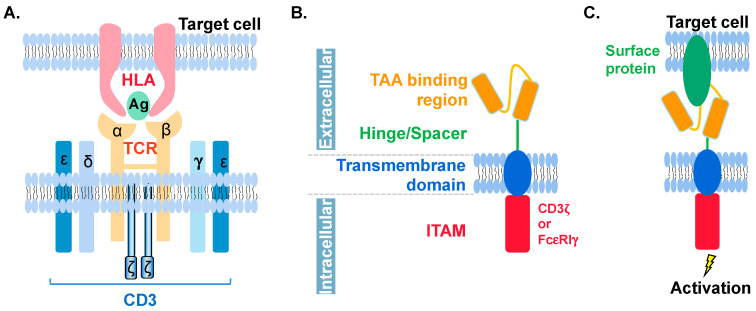
Schematic view of TCR and CAR molecules. (**A**) TCR (orange) on T cell is composed of α and β chains and binds to HLA (pink)-Ag (green) complex on target cell. CD3 (blue) has ε, ζ, γ, and δ chains. It serves as a co-receptor of TCR. (**B**) CAR is composed of the extracellular TAA binding region (orange), hinge or spacer region (green), transmembrane domains (blue), and the intracellular ITAM region (red) which is responsible for activating the cell once TAA binding region senses its target molecules. ITAM can be corresponding domains from CD3ζ or FcεRIγ. Detailed information can be found in Table 1. (**C**) CAR binds to target protein (green) on cell surface to trigger downstream activation. Noted that, neither HLA nor CD3 is required here.

Under physiological conditions, antigen recognition by T cells is accomplished by the TCR-CD3 complex. The TCR consists of α and β chains, the structure of which determines the antigen specificity of the TCR. CD3 contains four subunits (γ, δ, ε, and ζ) and functions as a co-receptor for the TCR (Figure 1A). CTL recognizes target cells through the binding of TCR to antigen-presenting human leukocyte antigen-I (HLA-I), triggering itself to secrete granzymes, perforins, and cytokines, such as interferon-γ (IFN-γ) and tumor necrosis factor-α (TNF-α), to kill the targets [7].

Engineered T cells that encode the exogenous TCR and that target tumor antigens are used in cancer treatment [8]. The current implementation of this strategy focuses on isolating TCRs with the best specificity and affinity through the design of molecular mechanisms to prevent the cross-reactivity of TCRs and reducing the mismatch between the α and β chains. TCR-T cell therapy has demonstrated significant anti-cancer effects against melanoma and sarcoma in small-scale clinical trials [9]. Another way to activate CD8^+^ T cells against cancer cells is through the introduction an artificial receptor with an extracellular region that is able to recognize cancer antigens and the intracellular fragment to initiate CD8^+^ T cell activation. In 1989, Gross and his colleagues introduced the concept of the chimeric antigen receptor (CAR) by fusing the antibody’s antigen-binding region, which is a single-chain fragment variable (scFv), with the CD3ζ chain or the intracellular portion of Fc receptor, FcεRIγ [10]. Thus, the basic structure of a CAR extracellularly to intracellularly consists of a tumor-associated antigen (TAA) binding region, a hinge or spacer region to provide flexibility to the TAA region, a transmembrane domain (TM) that enables CAR anchoring on the plasma membrane, and an immunoreceptor tyrosine-based activation motif (ITAM) to activate downstream signaling (Figure 1B,C) [11]. Detailed information on CAR will be discussed later in this review.

The best example of CAR-T application is CD19 CAR-T therapy. CD19 is a B-cell surface antigen molecule that is expressed by the majority of B-lineage-derived malignancies, including B-cell acute lymphoblastic leukemia (ALL), chronic lymphocytic leukemia (CLL), and non-Hodgkin’s lymphoma (NHL) cells [12,13,14]. Although targeting CD19causes B-cell dysplasia, it is tolerated by patients in the short term, and the removal of B cells may prevent the production of antibodies against CAR. CD19 CAR-T therapy has been successfully tested in clinical trials for the treatment of a variety of B-cell carcinomas, including ALL, CLL, and diffuse large B-cell lymphoma (DLBCL) [15]. It is encouraging to note that although these CAR-T therapies differ in CAR design, transgenic vectors, and manufacturing processes, they have all achieved very high complete response rates, particularly for ALL [16]. The advent of four FDA-approved CD19 CAR-T cell products, i.e., tisagenlecleucel, axicabtagene ciloleucel, brexucabtagene autoleucel, and lisocabtagene maraleucel, represents a breakthrough in the use of engineered T cells in hematological malignancies.

## 2. Adverse Events of CAR-T Therapy

Nevertheless, CAR-T has adverse effects that pose health risks to patients. Graft-versus-host disease (GvHD), cytokine release syndrome (CRS), and immune effector cell-associated neurotoxicity syndrome (ICANS) are the three most well-known problems observed upon CAR-T treatment. Allogeneic CAR-T cells made from healthy donors can attack the host’s normal tissue based on donor–recipient HLA mismatch, resulting in significant non-relapse-related morbidity and mortality [17]. In addition, rejection may lead to the clearance of allogeneic CAR-T cells [18,19,20,21]. While GvHD is an issue in allogeneic settings, many of the current applications use autologous products. Moreover, genetically engineered CAR-T cells can be made to avoid or reduce the risk of GvHD. One way to achieve this is to eliminate endogenous TCR expression based on gene editing [22,23,24]. After CAR-T cells are infused into the body, the recognition of target cells leads to the activation and proliferation of CAR-T cells, killing target cells and releasing a large number of cytokines, including IL-1, IL-2, IL-6, IL-8, IL-10, soluble interleukin 2 receptor-α (sIL-2Rα), sIL-6R, soluble glycoprotein130 (sgp130), IFN-γ, and granulocyte macrophage colony-stimulating factor (GM-CSF) [25]. Experiments have shown that IL-6 as a crucial mediator of CRS [26]. The released cytokines further activate host immune cells (e.g., T cells, B cells, monocytes, and macrophages) and non-immune cells (e.g., endothelial cells), resulting in the release of more cytokines from these host cells, creating a positive feedback effect that causes CRS to be featured as a systemic inflammatory response and that also causes severe damage to tissues and organs [14]. Some patients with ALL develop severe neurotoxicity after receiving CAR-T cell therapy. ICANS usually presents as toxic encephalopathy, with the earliest symptoms being decreased attention and speech and handwriting impairment. Other signs and symptoms include confusion, disorientation, agitation, aphasia, drowsiness, tremor, etc. [27]. One possible mechanism for the occurrence of ICANS in CD19 CAR-T therapy is the disruption of the blood–brain barrier (BBB), which may be linked to the fact that the pericytes of the brain express CD19 as well [28]. Moreover, CAR-T therapy may also lead to on-target/off-tumor toxicity, lymphodepletion-related toxicity, and allergic reactions. It is worth noting that the occurrence of the above-mentioned adverse reactions during the therapy is individual-specific. For example, in patients treated with CD19 CAR-T cells, CRS occurs in approximately 25% of patients, severe ICANS occurs in 12–42%, and non-relapse related death occurs in 1–2% [29]. Another concerning issue is that patients who have their therapy applied using CAR-T cells may relapse or develop other symptoms depending on individual immune status and disease. Long-term survival studies of CAR-T cell therapy have shown that some patients relapse within one year post treatment [27,30]. *CD19* mRNA damage resulting in the loss of its expression was observed in some patients. Additionally, the low efficacy and sustaining ability of CAR-T cells contribute to disease relapse [31]. A case of recurrent B-cell ALL was reported to demonstrate abnormal myeloperoxidase expression after CAR-T cell therapy [32].

Moreover, due to the high costs of production, storage, and transportation, the cost of CAR-T therapy is high. Currently approved anti-CD19 CAR-T cell products are valued at USD 373,000 (Axicabtagene Ciloleucel, Gilead/Kite Pharma) and USD 475,000 (Tisagenlecleucel, Novartis) per patient. Costs can reach roughly USD 1 million per patient after accounting for medical staff and hospitalization expenses [33]. Therefore, the financial toxicity of CAR-T therapy also limits its application.

## 3. Recognition and Killing of Cancer Cells by NK Cells

NK cells are also cytotoxic immune cells and represent a promising alternative to CAR-T cells. They are the first line of defense of the human immune system and were discovered by Ronald Heberman in 1975 [34]. NK cells account for approximately 10–20% of total peripheral blood lymphocytes and are mainly derived from bone marrow CD34^+^ lymphoid progenitor cells [35]. NK cells do not express specific antigen recognition receptors and are a third class of lymphocytes that are distinct from T cells and B cells. Currently, NK cells are defined as CD3^−^/CD56^+^ lymphocytes and can be divided into two cell subpopulations based on the level of CD56 expression: CD56^dim^ and CD56^bright^ [36]. The CD56^bright^ NK cell subpopulation accounts for about 10% of all NK cells, mainly accumulates in secondary lymphoid and non-lymphoid tissues, and secretes a large number of cytokines, including IFN-γ, IL-12, IL-15, and IL-18 to exert immunomodulatory effects. The CD56^dim^ NK cell subpopulation accounts for about 90% of all NK cells and has high FcγIII receptor (also known as CD16a) expression and high levels of granzymes and perforin-containing lytic granules.

**Figure 2:**
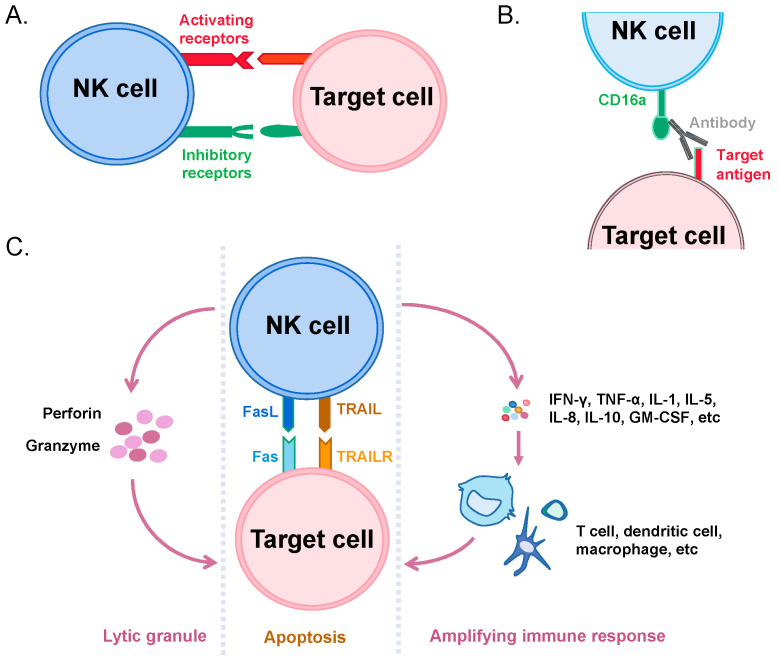
Cartoon illustration of NK cell activation and cytotoxicity. (**A**) NK cell can bind to target cell through both activating receptors (red) and inhibitory receptors (green). Its status is dependent on which signaling is dominant. (**B**) NK cell can also be activated through its CD16a binding to a cell-bound antibody. (**C**) Activated NK cells kill target cells through the release of lytic granules and FasL-Fas/TRAIL-TRAILR axis. NK cells also recruit other immune cells through the cytokine release to amplify the immune response against target cells.

NK cells can kill tumor cells and virus-infected cells at a very early stage. When the receptors that are involved in activating NK cells bind to their ligands on other cells, they initiate activation and exert their cytotoxic activity. The best-known activating receptor, NKG2D, binds to its ligand, which is normally absent in healthy tissues but is expressed on stressed or cancer cells such as hepatocellular carcinoma cells, myeloid leukemia, and ovarian cancer cells [37,38,39]. Additionally, activation signals also include integrins, killing receptors (e.g., NKp40, NKp30, and NKp44), and killer cell immunoglobulin-like receptors (KIRs) with a short cytoplasmic region, among others (e.g., NKp80, SLAMs, CD18, and CD2) [40]. Moreover, other ligand-receptor bindings result in the de-activation of NK cytotoxicity. The inhibitory signals mostly include receptors that recognize HLA-I such as Ly49s, NKG2A, and LLT1 and some molecules that are unrelated to HLA-I [41]. Among them, HLA-I-related inhibitory receptors play critical roles and can be generally classified into three types based on structure and function: KIRs with longer cytoplasmic domains, killer lectin-like receptors (KLRs), and leukocyte immunoglobulin-like receptors (LILRs) [42]. It is noteworthy that two forms of KIRs are present on NK cells, which have intracellular domains of different lengths. The ones with a shorter form are responsible for the activation of NK cells, and those with a longer form are inhibitory. The *KIR* gene, similar to the *HLA* gene, is inherited as a haplotype, and there are two different haplotypes, namely group A and group B. Group A haplotypes contained genes that encode 3DL3, 2DL3, 2DP1, 2DL1, 3DP1, 2DL4, 3DL1, 2DS4, and 3DL2. Group B haplotypes have genes that encode 3DL3, 2DS2, 2DL2, 3DP1, 2DL4, 3DS1, 2DL5, 3DS5, 2DS1, and 3DL2. Among these, 2DL4, 2DS4, 2DS2, 2DL4, 3DS1, 3DS5, and 2DS1 serve as activating receptors, while 3DL3, 2DL3, 2DL1, 3DL1, 3DL2, 3DL3, 2DL2, 2DL5, and 3DL2 are inhibitory ones [43]. Therefore, the status of NK cells is determined by the competition between the activating and inhibitory receptors (Figure 2A). In addition, NK cells express CD16a on the surface to bind to target cell-bound antibodies, activating the cytotoxic activity of NK, a process known as antibody-dependent cell-mediated cytotoxicity (ADCC) (Figure 2B) [44].

Activated NK cells mainly kill target cells through two mechanisms. The first is through the release of cytotoxic particles such as perforins and granzymes through exocytosis, which induces target cell lysis or apoptosis [45]. The second is through the expression of Fas (also known as CD95) ligand (FasL) and TNF-related apoptosis-inducing ligand (TRAIL) molecules upon its activation, which induce apoptosis in Fas (CD95)-expressed and TRAIL receptor (TRAILR)-positive target cells through a cascade reaction of endogenous enzymes [46]. Moreover, activated NK cells secrete cytokines to amplify the immune response. A variety of cytokines, such as IFN-γ, TNF-α, IL-1, IL-5, IL-8, IL-10, and GM-CSF, were produced and secreted [47]. These cytokines bind to other immune cells, leading to their activation and proliferation (Figure 2C) [48]. For example, NK cells are early and potential producers of IFN-γ and have many effects on immune response, including the induction of antigen-presenting cells by HLA-II molecules, the activation of myeloid cells, and the induction of T helper 1 (Th1) cells [49].

## 4. Immunotherapeutic Applications of NK Cells

Unlike in CAR-T therapy, where the HLA-I in the recipient host leads to GvHD, HLA-I has a dominant-negative regulatory function on NK killing activity. Moreover, tumor cells are mostly down-regulated with HLA-I expression, which, in turn, favors NK killing [50]. Thus, transfused NK cells do not need to be collected from patients or specific HLA-matched donors, making them a good substitute for T cells. NK cell-based immunotherapy can be divided into two pathways: the activation of NK cells through the activating receptors and the blockade of inhibitory receptors. These methods are available through the following means: (1) Cytokine therapy: Patients are infused with cytokines such as IL-2 and IL-15 to activate their own NK cell activity in vivo [51]. (2) Adoptive cell therapy (ACT) of NK cells: Patients are infused with healthy, activated autologous or allogeneic NK cells, in which the returned NK cells are treated in vitro with the activation of cytokines such as IL-2 and IL-15 [52]. (3) Genetically engineered NK cell therapy: The infusion of genetically modified autologous or allogeneic NK cells, such as CAR-modified NK cells (CAR-NK), into patients [53]. (4) Monoclonal antibody therapy: Depending on the targets of the antibodies, there are two categories: i. Therapeutic antibodies targeting tumor-associated antigens, such as Cetuximab targeting the epidermal growth factor receptor (EGFR) and Rituximab targeting CD20. These antibodies can be used for therapeutic purposes through NK cells to induce ADCC [54]. ii. The use of antibodies that target NK cell inhibitory receptors directly, such as Monalizumab, which targets NKG2A and activates NK cells to kill tumor cells [55] and Lirilumab, an inhibitory KIR receptor inhibitor that specifically binds to the inhibitory KIR2DL1, -2, and -3 receptors with high affinity (and activates KIR2DS1 and -2 receptors), blocking KIR interaction with HLA-C and enhancing the NK cell killing of tumor cells [56]. (5) Approaches using haploidentical donors with KIR-HLA mismatch: As mentioned earlier, KIR-HLA matching is important to inhibit NK cytotoxicity. Tumor cells can be killed by autologous NK cells due to this mismatch between donor inhibitory KIRs and recipient HLA molecules. Clinical studies have shown that NK cells from the haploidentical donors with unmatched KIRs promoted the therapeutic effect and improve the prognosis of lymphoid disease patients [57]. (6) NK cell engagers: Engagers are generally bispecific or trispecific antibodies that target both tumor cell antigens and NK cell activation receptors, bringing NK cells and tumor cells together and triggering NK cells to kill tumor cells (e.g., engagers developed by Affimed Heidelberg, Germany) [58].

## 5. Comparison of Biology in CAR-NK and CAR-T

Similar to T cells, NK cells were genetically engineered to encode CARs that recognize tumor antigens. CAR-NK has become one of the most popular directions of research in recent years. The sources, current development, advantages, problems, and clinical status of NK cells in CAR-NK therapy will be summarized below.

Compared to CAR-T therapy, CAR-NK has improved security. As far as we know, no reports of CRS caused by CAR-NK therapy have ever been found, possibly due to activated NK not releasing IL-6, a critical cytokine believed to induce CRS [59]. In addition, NK cells can target tumors without antigen pre-sensitization or HLA matching, thus resulting in no GvHD response, meaning that they can be made into an off-the-shelf product and frozen for long-term storage [60].

CAR-NK can recognize cancer cells through several mechanisms. Besides through the CAR recognition of tumor surface antigens, CAR-NK cells can also recognize cancer cells through a variety of previously described receptors, such as natural cytotoxic receptors (NKp46, NKp44, and NKp30), NKG2D, and DNAM-1 (CD226), conferring a higher targeting efficacy compared to CAR-T cells. In addition, NK cells can be guided by antibodies through their CD16a [44,61]. Clinical trials have shown that CAR-T cells cannot eliminate highly heterogeneous cancer cells, but CAR-NK cells are effective in killing residual tumor cells that may change their phenotype after long-term treatment [62]. Indeed, it was observed that cancer stem-like cells are hidden in cancer cells due to the lack of CAR-directed antigens, leading to the ineffectiveness of CAR-T therapy [63]. However, the incidence of tumor evasion is limited in CAR-NK therapy because CAR-NK cells have both a CAR-dependent and CAR-independent targeting capacity.

Moreover, T cell immune checkpoints have impacts on CAR-T therapy. Programmed cell death protein 1 (PD-1) is an immune checkpoint receptor that is expressed on the surface of T cell. Its binding to the PD-L1 expressed on target cell sends an inactivating signal to the T cell, thus inhibiting the T cell’s immunoactivities. Unfortunately, many types of cancer cells are known to express high levels of PD-L1, resulting in cancer cell survival upon T cell engagement. This could be overcome by targeted therapy. Inhibitors of the PD-1-PD-L1 axis have been shown to achieve good effects clinically, e.g., Nivolumab (PD-1 antibody) in the treatment of relapsed or refractory classical Hodgkin’s lymphoma (CHL) [64]. In the case of CAR-T cells, the PD-1-PD-L1 axis suppresses their cytotoxicity and thus protects tumor cells from being killed [65], creating a challenge for CAR-T therapy. On the contrary, NK cells have very low surface PD-1 expression. Thus, they are relatively less affected by tumor-expressed PD-L1 [66]. Given these advantages, CAR-NK cell therapy has been shown to kill a variety of blood and solid tumor cells, such as those in triple-negative breast cancer, bladder, and lung tumors, in preclinical and clinical trials [67].

Finally, the durability of CAR-T and CAR-NK cells is different. It is known that CAR-T cells can be sustained in the patient for a few years, while CAR-NK cells only several months [68]. There seems to be a debate about the durability of these immune cells. On the one hand, longer persistence means effectiveness can be sustained for a longer period of time. On the other hand, it may lead to safety concerns of allogenic cytotoxic immune cells existing in the body.

## 6. Molecular Features of CAR in CAR-NK and CAR-T

CAR should not only ensure the specific recognition of target cells, but also achieve the activation of NK cells. The design of the CAR structure and its transduction are critical to the therapeutic efficacy of CAR-NK. The CAR of NK follows the basic structural framework and signal transduction of CAR-T. While early CAR-NK constructs were based more on the costimulatory domain involved in T cell activation, some later studies found that DAP10, DAP12, and 2B4 play an important role in NK activation and that these intracellular signaling adapters mediate NK cell activation upon binding to their respective upstream receptors [69,70,71].

The single-chain antibody fragment responsible for tumor antigen binding determines the specificity of CAR-T and CAR-NK cells. It has also been shown that the order of heavy (VH) and light (VL) chains does not affect the expression level of CARs on T cells that can target tumor-associated antigens [72]. So far, most prefer the VH-VL direction rather than the VL-VH direction for this region [73]. In addition, the cells can be equipped with multiple single-chain antibodies, thereby expanding the antigen recognition capacity of CAR effector cells [74].

The hinge region is the extracellular structural region that connects single-chain antibody units to the transmembrane structural domain, which normally maintains the stability required for robust CAR expression and activity in effector cells, providing flexibility to the single-chain antibody fragment. Most CARs use derivatives of the extracellular domain of CD8α or CD28 as the hinge region [75], while others use the Fc portion of IgG1 or IgG4 or the CH2/CH3 domain of the Fc portion as the hinge region [76]. The length of the hinge region was found to be adjustable to accommodate antigen recognition. Experiments have shown that more efficient cytokine production and the proliferation of CAR-T cells that contain a short 12 amino acid (aa) spacer compared to those with intermediate (119 aa) or long (229 aa) spacers. The shorter the spacer region, the higher the cytokine production, the faster the proliferation of CAR-T cells, and the better the in vivo persistence and anti-tumor effect [77].

The TM domain connects the extracellular structural domain of the CAR to the intracellular activation signaling structural domain. The most common TM portion of the CAR molecule used in NK cells is derived from CD3ζ, CD8, or CD28, but others, such as NKG2D, 2B4, and DNAM1, have also been used [78].

The intracellular signal region determines the strength of the activation signal and directly affects the killing effect. First-generation CAR-NK cells, such as CAR-T cells, only contain CD3ζ signaling and consist of three ITAMs. Second- and third-generation CAR-NK carry one and two additional co-stimulatory signals, respectively, and co-stimulatory molecules are usually derived from the CD28 family (CD28 and ICOS), the TNFR family (4-1BB, OX40, and CD27), or the SLAM-related receptor family (2B4) [79]. Most published CAR-NK clinical trials have used a second-generation CAR-NK construct that enhances activity by adding IL-15 expression and inducing Caspase9 [53]. Strong activation signals are important to induce an effective antitumor response but may also lead to the rapid failure of effector cells [73], so combinations of co-stimulatory domains can be used to calibrate the desired immune cell response. Compared to 4-1BB-based CARs, CD28-based CARs exhibit a faster effector profile, inducing higher levels of IFN-γ, granzyme B, and TNF-α, resulting in typically greater cytokine release from CD28-containing CARs, which also leads to activation-induced cell death (AICD) [80,81]. In contrast, 4-1BB-CD3ζ signaling preferentially induces memory-related genes and sustained antitumor activity [82], possibly because the 4-1BB structural domain ameliorates the T-cell depletion induced by the CD28 structural domain.

**Figure 3:**
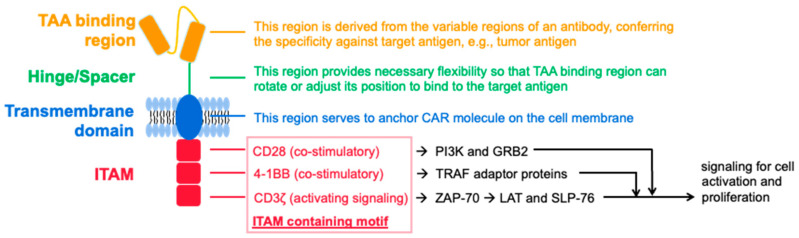
Cartoon illustration of the principle of the construction of CAR molecule for NK cells. The functions of different regions are indicated on the right. Intracellular events are briefly summarized and shown in black.

Table 1 summarizes the molecular composition and available components of CAR in CAR-T and CAR-NK. A graph illustrating the principle of the construction of the CAR molecules for NK cells can be found in Figure 3. As shown, CAR molecules for both T and NK cells share similar architectures as well as motifs for each region [83,84,85].

## 7. Source and Preparation of NK or CAR-NK Cells for Clinical Use

The preparation and application of NK cell therapy are similar to other immune cell therapies, which can be summarized into three steps: cell acquisition, amplification and production, and clinical use. An important issue in CAR-NK therapy is the source of NK cells, which mainly includes NK cells isolated from peripheral blood (PB), umbilical cord blood (UCB), or the in vitro cultured NK cell lines [68].

PB-derived NK cells (PB-NKs in short) have the advantages of safety and good cytotoxicity but have low cell purity and are difficult to mass expand. Moreover, cryopreservation can affect cell activity, posing a great challenge for large-scale production [86]. UCB-derived NK cells (CB-NKs or UCB-NKs for short) can be differentiated and expanded with good clinical outcomes but may have the risk of incomplete differentiation, limited lethality, and tumorigenicity [87]. In addition, for PB-NK and CB-NK cells, there may also be differences in the NK cells per blood unit, e.g., the type of KIR expression [88]. NK cells have many cell lines of persistent passage, including NK-92, NKG, YT, NK-YS, HANK-1, YTS, and NKL [89]. The NK-92 cell line, a strain of IL-2-dependent NK cells derived from the peripheral blood mononuclear cells of a 50-year-old white male with aggressive NHL, is widely used in current clinical trials [90]. The major advantages of the NK-92 cell line over primary NK cells are that it does not involve a sorting and purification step, the consistency of the cell population obtained by in vitro expansion, and the low expression of the killing inhibitory receptors on its surface, resulting in superior killing ability. However, the disadvantage of the NK-92 cell line is also clear: these cells are aneuploid and must be irradiated before being administered to patients to avoid becoming a new source of cancer cells after treatment [91]. The irradiation limits their ability to expand and persist in vivo, reducing antitumor efficacy [92]. Preclinical studies and clinical trials of cell therapy have shown that improved CAR-T cell persistence leads to better therapeutic efficacy [93,94]. Similar studies on NK cells have also shown that persistence in preclinical in vivo models correlates with better tumor killing ability [67]. Therefore, this limited expansion and persistence after administration to patients may explain the limited efficacy of NK-92 cells in some clinical trials.

In current clinical studies, NK cells are used in large amounts, between 5 × 10^6^ and 5 × 10^7^ per kilogram of body weight [95], and the amount of NK cells in normal human PB and UCB is far from adequate for clinical proposes. A typical method for generating such large numbers of allogeneic NK cells is to enrich NK cells from donor-derived leukocyte isolation products followed by in vitro expansion. By the immunomagnetic bead removal of T and B cells and the enrichment of CD56^+^ cells, different protocols have been established to expand NK cell populations, including the use of cytokines, feeder cells, membrane particles, or a combination of these approaches. For example, culturing over 14 days using a combination of the cytokines IL-2 and IL-18 amplified NK cells from CD3-depleted PBMCs [96]. Another approach using autologous PBMC in the presence of recombinant OKT3 and IL-2 led to the large-scale expansion of good manufacturing practice (GMP)-compliant NK cells after a 14 days of culturing and showed cytolytic activity against tumor cells [97]. In the in vitro NK expansion of UCB-derived CD56^+^ cells, it was reported that UCB-derived mononuclear cells (MNCs) stimulated with IL-2, group A Streptococcus, and zoledronate for three weeks produced NK cells with an average purity of 95% and expanded by 1561-fold. This feeder layer-free approach also reduces the risk of introducing other cell contaminants into the final product [98].

The purity of these products is particularly important for their use in the allogeneic environment, as residual T and B cells can lead to GvHD and passenger B lymphocyte-mediated complications, respectively [78]. Moreover, this preparation strategy has a high time cost, which not only affects the recovery of NK cells, but also the vitality and potency of NK cells. Obtaining sufficient NK cells from a single leukocyte isolate is difficult due to the low recovery rate, not to mention the limited availability of donor-derived leukocyte isolates. Therefore, how to isolate and prepare high-quality and high-purity NK cells has become an urgent problem to be solved in clinical application.

In vitro differentiation from a large amount of progenitor cells can be used to circumvent this issue. During the differentiation from stem cells to NK, the cells go through an intermediate state of CD34^+^ hematopoietic stem cells (HSCs). HSCs are responsible for the production of all of the blood cells in the body, including innate immune cells (monocytes, macrophages, granulocytes, NK cells, and dendritic cells) and adaptive immune cells (B lymphocytes and T lymphocytes) [99]. Traditional sources of HSC include adult bone marrow and the umbilical cord of newborns. Unfortunately, adult or umbilical HSC amplification protocols are prone to failure and differentiation [100,101]. Alternative or complementary methods to achieve large numbers of HSCs include the use of induced pluripotent stem cells (iPSCs), which are highly scalable due to the strict culture conditions of iPSCs [102]. One method is to co-culture using mouse bone marrow stromal cells (e.g., S17 and M2-10B4) for 21 days to obtain CD34^+^ hematopoietic precursors, after which CD34^+^ cells are sorted and then differentiated into NK cells [103]. Another method is the formation of embryoid bodies (EBs) or spin-EBs. The iPSCs are resuscitated and passaged 2–3 times, and then the cell clusters are digested into single cells using specific digestive enzymes such as Accutase. Single cells are differentiated by specific media to form anthropoid EBs, and some laboratories have adopted the spin-EB form to improve differentiation efficiency [104]. After that, EBs/Spin-EBs continue to be cultured in differentiation medium to obtain CD34^+^ HSC and continued to differentiate with differentiation medium to obtain NK cells. NK cells are FACS analyzed and screened by markers on the surface (such as CD16a, CD56, CD43, NKp46, NKp44, NKG2D), and the resulting NK cells are amplified and frozen for long-term storage. The cells can then be resuscitated and injected into the patient when needed [78,105,106,107,108,109,110].

Nevertheless, this method also has its drawbacks. The NK cells obtained in the form of EBs express high levels of KIRs, which limits their application to certain HLA-typed recipients [111]. Clinically, KIR-based therapeutic interventions are needed to improve clinical outcomes, and, in addition, to using NK cells from KIR-HLA mismatched donors, a blocking anti-KIR antibody that binds KIR2DL1/L2/L3 has been used in clinical trials to reduce the suppression of NK cell activity by HLA-C alleles [112]. Alternatively, there have been successful reports of KIR-free NK cells being obtained. H1 embryonic stem cells (ES) were co-cultured with feeder mouse OP9 cells without EB morphology to directly obtain CD34^+^ HSC cells, which were then co-cultured with OP9 cells expressing Delta-like-1 (DLL1), a Notch ligand that inhibits B-cell development, to promote NK cell development into human cord blood CD34^+^ cells [113]. DLL1 can enhance the proliferation of primitive hematopoietic progenitor cells in vitro. The NK cells obtained by this method do not express KIRs on their surface, which makes them recipient HLA genotype-independent [114]. Theoretically, these HLA-unrestricted NK cells could serve as a universal “off-the-shelf” source of NK cells for many recipients. More and more efforts using matrix (such as matrigel, laminin 521, and laminin 511)-based culturing systems rather than feeder cell-based ones are being carried out [115].

CARs need to be added to CAR-NK cells. For NK cells derived from the differentiation of stem cells, there are two approaches. One is to add CARs onto the stem cells and to then differentiate them into CAR-expressing NK cells. The other is to introduce CAR into the differentiated NK cells, similar to the process in primary NK cells or NK cell lines. The problem is that NK cells are heterogeneous and hard to transduce, transfect, or nucleofect directly in vitro, which means that the cost of obtaining such CAR-NK cells could be high. The NK sources obtained from ES differentiation are relatively uniform and have better transduction efficiency. However, it may be important to first check whether the integrated CAR affects NK differentiation or not, which is not an issue for engineering on NK directly.

The stability of gene transduction is the prerequisite to achieving the stable expression of CAR in effector cells. Currently, CAR-NK transduction techniques that are commonly used include viral and non-viral vectors, among which commonly used viral vectors include retrovirus, lentivirus, and adeno-associated virus (AAV) [73]. In terms of preclinical studies, 21 studies used second-generation viruses, and six studies used third-generation lentiviruses to generate CAR-expressing NK cell lines (17 unknown). Among the primary CAR-NK cell studies, five studies used third-generation lentiviruses, and seven studies used second-generation lentiviral vectors (two unknown). In a recent Phase I clinical trial, CD19 CAR-NK cells transduced by retroviruses were used to treat CD19^+^ NHL and CLL. In this study, 73% of patients responded, and seven out of eight patients achieved complete remission. In addition, the response was rapid up to 30 days after CAR-NK infusion at all dose levels. Amplified CAR-NK cells could still be detected after one year of follow-up. CAR-NK DNA copy numbers in PB remained stable for up to one year after transfusion, and these results are the first indication that retroviral-transduced CAR-NK cells can survive for a long time in vivo [53]. Different kinds of retroviruses are used to produce CAR-NK cells. The RD114α retrovirus was more efficient in transducing primary NK cells than the γ retrovirus and lentivirus. Although stable CAR expression can be obtained in NK cells for long periods of time using different retroviruses, the safety of retroviral systems remains a concern, especially when compared to safer lentiviruses. During integration into the host genome, simple retroviruses (e.g., MLV) typically have about 20% of their infection events occur at the 5′-end of the transcription unit and have a preference for CpG islands, and near the DNase I hypersensitive site [116]. Lentiviral vectors are mostly integrated to sites far from the transcription start point [117].

Commonly used non-viral vectors include the sleeping beauty and PiggyBac transposons. The DNA or mRNA-based methods use liposome-mediated or nucleoporation-based approaches to be delivered into the cells. The electroporation of CAR-encoded mRNA is a rapid and effective but a short-lived method. In general, the mRNA transfection efficiency of amplified or activated NK cells is much higher than that of freshly isolated NK cells [118]. Since the synthesis of mRNA is GMP compliant and electroporation can be performed in a clean room, it is feasible to generate GMP compliant CAR-NK by mRNA electroporation. However, the main drawback of this approach is the short window of CAR expression. Studies have found that after electroporation, CAR-NK cells should be infused back into the patient within seven days [73,119].

Transposon-based systems can efficiently introduce CAR transgenes at a predetermined location, an important advantage that conventional methods do not have. They are introduced into NK cells mainly by electroporation and are then integrated into the host genome by transposase. Two studies have applied transposon systems to generate CAR-NK cells: one used NK-92-MI cells, and the other study transfected transposons into iPSCs that then differentiated into NK cells. After enrichment, anti-mesothelin CARs were stably expressed on iPSC-derived NK cells and functioned in ovarian cancer mouse models.

**Figure 4:**
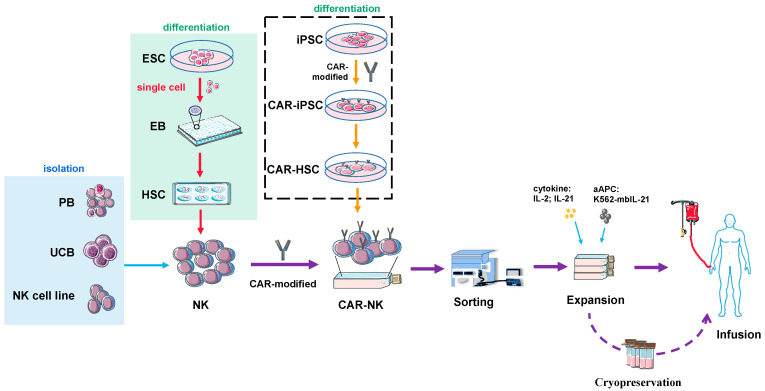
Flow chart of CAR-NK therapy from preparation of NK cells to infusion of patients. NK cells can be either isolated and expanded from primary sources such as PB and UCB or differentiated from ESC or iPSC. CAR molecules are introduced directly into NK cells or at the beginning of differentiation of iPSC. The prepared CAR-NK cells are sorted and further expanded using either cytokine stimulation or an APC cell (K562-mbIL-21). CAR-NK cells can also be cryopreserved for future use.

As for the differentiation and expansion protocols, different laboratories have slightly different media formulations and operation methods. Supplemental Table S1 summarizes and compares seven aspects, including the culture of ES/iPSC, the differentiation of ES/iPSC into HSC, the differentiation of HSC into NK cells, the amplification of NK cells, the selection of antibodies for phenotype identification, and in vitro and in vivo experiments [120,121,122,123,124,125]. In addition, there are also biological companies that offer specialized kits for the differentiation of HPSC into NK, such as the StemSpan™ NK Cell Generation Kit for stem cells, which includes components for the differentiation of CD34^+^ cells isolated from cord blood or bone marrow to NK cells. NK cells expand very slowly in vitro. At present, various small factors are added to promote NK cell proliferation, such as IL-21 and IL-15. IL-15 plays an important role in stimulating NK cell expansion and cytotoxic functions [126]. It shares the γc receptor subunit with IL-2, and both interleukins induce the differentiation of T cells, B cells, and NK cells as well as promote the proliferation of T cells, B cells, and NK cells [127]. At present, IL-15 is often used in combination with IL-2 to amplify T cells or NK cells in vitro [128]. It is able to induce lymphokine-activated killer cells activity and also stimulates IFN-γ production by NK cells together with IL-12 [129]. Without the addition of any exogenous factors in vivo and in vitro, the expression of membrane-binding protein IL-15 in the human PB-NK cell membrane can improve the survival and amplification ability of NK cells and can further enhance the ability to kill hematological malignancies and solid tumors [130]. IL-21 is another common γ-chain cytokine that is essential for the maturation and proliferation of NK cells [131,132]. The in vitro assays showed that IL-21 can lead to higher cytotoxicity in NK cells through the upregulation of IFN-γ and granzyme [133]. In addition, irradiated K562 has also been utilized, with these cells acting as artificial antigen-presenting cells (aAPCs) to express membrane-bound IL-21 (mbIL-21) and other stimulatory ligands (e.g., 4-1BBL or NK40L) to stimulate prolonged and large-scale expansion of NK cells [134]. Figure 4 illustrates the experimental flow chart discussed above.

## 8. Current Clinical Trials of CAR-NK

Several clinical trials of tumor cell immunotherapy with NK cells have been conducted worldwide, and CAR-NK cell therapy has shown preliminary clinical significance. In addition to its effectiveness in blood cancers, NK cells are being used as an important strategy to address solid tumors that CAR-T fails to address. For example, data from a phase I/IIa trial of CAR-NK cell therapy in 11 patients with relapsed/refractory NHL or CLL were published. Of these 11 patients, eight (73%) responded to treatment, and seven of them achieved complete remission after treatment, meaning they were free of signs of disease at a median follow-up of 13.8 months. The majority of patients responded significantly within 30 days of receiving the cell infusion, demonstrating progressive efficacy, and the durability of this treatment was confirmed within one year of the infusion (NCT00505245).

The safety of CAR-NK cell infusion has been demonstrated by the absence of serious adverse events during treatment and follow-up of patients (clinicaltrials.gov, accessed on 1 January 2020). Fate Therapeutics is developing an off-the-shelf, iPSC-derived CAR-NK cell therapy known as FT596, which has three anti-tumor modalities (expressing CD19 CAR, novel high-affinity non-cleavable CD16 Fc receptor, and IL-15RF) (NCT04245722). In September 2019, FT596 was approved for clinical studies by the FDA in less than one year, and on 2 April 2020, the first clinical studies of FT596 using patient administration were completed in BCL and CLL. As of 11 October 2021, 10 patients continue to achieve sustained remission, with three patients sustaining complete remission for at least six months after treatment initiation; two patients achieving six months of complete remission followed by disease progression; and one patient experiencing disease progression by six months. In addition, FT596 therapy has demonstrated good tolerability, with no dose-limiting toxicity and no ICANS or GvHD at any level detected in the trial. Two cases of low-grade CRS were reported in the trial (clinicaltrials.gov). The latest results from Fate Therapeutics’ NK cell therapy FT516 showed that eight of the 11 patients treated with FT516 achieved remission after three months of treatment, including six patients in complete remission (NCT04023071). However, after six months of treatment, three of the eight patients who were in remission experienced a recurrence of the disease or required additional anti-cancer therapy. No CRS, ICANS, or GvHD of any grade has been reported. Qihan Biologics’ development of engineered hiPSC-derived allogeneic NK cells is emerging as a promising safe and effective off-the-shelf cell therapy drug, with genetically engineered NK cells that are significantly enhanced in functionality and have significant killing ability against hematologic and solid tumors [135]. As of August 2022, there are 791 CAR-T therapy clinical trials that are registered, mostly in the US, China, and European countries (clinicaltrials.gov). Compared to CAR-T therapies, registrations for CAR-NK trials started later, with only 28 registrations (Table 2). The diseases targeted by CAR-NK therapy are mostly solid malignancies, such as pancreatic, ovarian, and prostate cancers. Most clinical trials utilize allogeneic NK cells, mainly from healthy donors or NK cell lines, such as NK92 (Table 3) [136].

Practically, immunotherapy using allogeneic cell sources can cause reduced efficacy because of the rejection of imported NK cells by the patient’s immune system. Current strategies in the field to address this issue are as follows: The first strategy is more effective means of lymphocyte clearance. It has been shown that in order for the imported cell therapy to proliferate in the patient’s body, the existing lymphocytes in the patient’s body need to be removed using chemotherapy or other means before the cells are imported, thus providing room for the imported cell therapy to proliferate [137]. For example, UCART19, developed by Allogene, uses gene editing to knock out the gene for cells to express CD52. This makes these cells resistant to alemtuzumab, a monoclonal antibody that kills T cells, by binding to CD52. Combining alemtuzumab with chemotherapy allows for the more effective clearance of mature T cells from the patient and maintains the host’s mature T cells at low levels. At the same time, allogeneic CAR-T therapies can still work [138]. The second strategy is to reduce the immunogenicity of allogeneic cells. Experience with organ transplantation and HSC transplantation has shown that the immune rejection of the graft can be substantially reduced if the HLA phenotype of the donor is similar to the HLA phenotype of the host [139]. Based on this experience, it is theoretically possible to generate homozygous cells by discovering donors with specific HLA phenotypes, constructing a donor cell bank from which donor cells matching the patient can be selected, or by designing iPSCs without the surface expression of classical HLA class I proteins through endogenous B2M gene knockout and HLA-G1 (or HLA-E) knock-in, which can then be differentiated into NK cells for therapeutic use [140].

A final risk worth noting is the application of retroviruses. CAR-T/NK therapies typically use lentiviruses to introduce CAR genes into cells [141], and the use of such systems relies on well-defined viral packaging methods. To date, there has been no mention of how this randomized integration affects clinical outcomes for patients. These genes that are integrated with the help of the lentivirus may insert into oncogenes or tumor suppressors, altering the transcript levels or sequences of the original genes, causing a potential oncogenic risk that cannot be avoided in the use of lentiviral systems [142]. The use of clones that are integrated into specific regions by knock-in [143] or by testing monoclonal integration sites to select for non-oncogene integration may provide an effective hedge against this risk. In addition, there may be differences in the copy number of CARs integrated into different cells, which may affect the therapeutic effect. Lentiviral-integrated gene sequences may also be subject to chromatin-dependent gene silencing, with transgene expression levels decreasing over time [144]. This silencing has detrimental effects on cancer therapy.

## 9. Conclusions and Prospects

This review discussed CAR-T and CAR-NK therapies as well as the preparation and clinical progress of CAR-NK. Compared to CAR-T, CAR-NK has advantages in terms of NK cell source and side effects. Preclinical studies have shown that CD19-CAR NK cells have a high response rate to hematological tumors and are easy to manufacture [137]. In addition to CD19, the clinical studies of CAR-NK cells in lymphomas and leukemia have also targeted CD7 (NCT02742727) and CD33 (NCT02944162). Several clinical trials of CAR-NK cells for hematological malignancies are currently underway [145].

CAR-NK cells have their unique advantages compared to CAR-T cells, but there are still some challenges, including the time to prepare CAR-NK cells; the cost of the product, including the costs of preparation, storage, and transportation; and the use of animal-derived products during preparation. Although allogeneic NK cells have been shown to have potent anti-AML activity, their efficacy in treating solid tumors is limited [146]. One of the main reasons is that the adoptive transfer of primary NK cells usually last for a relatively short time in vivo, limiting their antitumor efficacy. Unlike autologous CAR-T cell-based therapies, which can persist and maintain function for months or years [147], allogeneic NK cells typically survive for much less time in an adoptive transfer environment. IPSC-derived NK cells are technically difficult and still face regulatory challenges for safety and clinical efficacy; they also have potential oncogenic risks and have been studied extensively in terms of epigenetic modification, chromatin organization, and metabolic reprogramming to inhibit their oncogenic potential.

Finally, to develop CAR-NK as a safe, effective, and “off-the-shelf” cancer immunotherapy, there is a general agreement in the community that the future can be optimized in the following ways: The first step is to simplify and optimize the CAR-NK manufacturing process and the efficiency of “off-the-shelf” product recovery after freezing. The second step is to improve CAR design for optimal NK cell activation and cytotoxicity and to reprogram CAR-NK cells to overcome tumor suppression and escape. The third step is to develop recombinant CAR-NK cells with memory properties in vivo for the long-term monitoring of tumors. Finally, enhanced CAR-NK cell infiltration into solid tumors should be optimized to improve the non-specific natural killing function of CAR-NK cells in combination with CAR-NK cell-derived specific killing. Based on the excellent anti-tumor lineage of NK cells, it is believed that solving these problems will most likely create a new breakthrough in tumor therapy armed with CAR modification.

## Figures and Tables

**Table 1:** Comparison of CAR molecules between CAR-T and CAR-NK.

Domains	CAR-T		CAR-NK
TAA binding region	VH-VL, VL-VH		VH-VL, VL-VH, VH-only
Hinge domain/spacer	Non IgG-based:	CD8a, CD28	CD8a, CD28, DAP12, IgG4, IgG2 CH2-CH3, IgG1 CH2-CH3, IgG4 CH2-CH3
	IgG-based:	IgG1, IgG4	
Transmembrane	CD3ζ, CD28, ICOS, CD8a, CD4		CD3ζ, CD28, CD8a, 4-1BB, DAP12, TCR ab, CD28-CD3ζ, FceRI*γ*, murine CD3ζ
ITAM	e.g., CD3ζ, CD28, 4-1BB, CD27, ICOS, OX-40, MYD88, CD40, KIR2DSS2		e.g., CD3ζ, CD28, 4-1BB

Note that, domains of CAR molecules come from corresponding regions of the genes listed here. Examples of motifs used as ITAM for CAR molecule are shown.

**Table 2:** Number of CAR-therapy clinical trials worldwide.

Region Name	China	United States	Japan	Europe	Other Countries and Regions
CAR-T	447	240	10	59	35
CAR-NK	18	5	0	0	5

**Table 3:** Clinical trial summary of CAR-NK therapy.

Clinical Trial	NK Source	Interventions	Conditions	Status	Locations	Phase
NCT04887012	unpublished	Biological: anti-CD19 CAR-NK	B-cell Non Hodgkin Lymphoma	Recruiting	2nd Affiliated Hospital, School of Medicine, Zhejiang University Hangzhou, Zhejiang, China	Phase 1
NCT05213195	unpublished	Drug: NKG2D CAR-NK	Refractory Metastatic Colorectal Cancer	Recruiting	The First Affiliated Hospital, Zhejiang University Hangzhou, Zhejiang, China	Phase 1
NCT05215015	unpublished	Biological: Anti-CD33/CLL1 CAR-NK Cells	Acute Myeloid Leukemia	Recruiting	Wuxi People’s Hospital Wuxi, Jiangsu, China	Early Phase 1
NCT05194709	unpublished	Biological: Anti-CAR-NK Cells	Advanced Solid Tumors	Recruiting	Wuxi People’s Hospital Wuxi, Jiangsu, China	Early Phase 1
NCT04639739	unpublished	Biological: anti-CD19 CAR NK	NHL	Not yet recruiting	Department of Hematology, Xinqiao Hospital ChongQing, Chongqing, China	Early Phase 1
NCT03692767	unpublished	Biological: Anti-CD22 CAR NK Cells	Refractory B-Cell Lymphoma	Unknown		Early Phase 1
NCT03690310	unpublished	Biological: Anti-CD19 CAR NK Cells	Refractory B-Cell Lymphoma	Unknown		Early Phase 1
NCT05008575	unpublished	Biological: anti-CD33 CAR NK cells Drug: Fludarabine Drug: Cytoxan	Leukemia, Myeloid, Acute	Recruiting	Department of Hematology, Xinqiao Hospital Chongqing, Chongqing, China	Phase 1
NCT04324996	unpublished	Biological: NK cells, IL15-NK cells, NKG2D CAR-NK cells, ACE2 CAR-NK cells, NKG2D-ACE2 CAR-NK cells	COVID-19	Recruiting	Chongqing Public Health Medical Center Chongqing, China	Phase 1 Phase 2
NCT03692637	peripheral blood	Biological: anti-Mesothelin Car NK Cells	Epithelial Ovarian Cancer	Unknown		Early Phase 1
NCT03415100	peripheral blood	Biological: CAR-NK cells targeting NKG2D ligands	Solid Tumours	Unknown	Third Affiliated Hospital of Guangzhou Medical University Guangzhou, Guangdong, China	Phase 1
NCT03692663	unpublished	Biological: anti-PSMA CAR NK cells	Castration-resistant Prostate Cancer	Unknown		Early Phase 1
NCT05008536	unpublished	Biological: Anti-BCMA CAR-NK Cells Drug: Fludarabine Drug: Cytoxan	Multiple Myeloma, Refractory	Recruiting	Department of Hematology, Xinqiao Hospital Chongqing, Chongqing, China	Early Phase 1
NCT03940820	unpublished	Biological: ROBO1 CAR-NK cells	Solid Tumor	Recruiting	Radiation Therapy Department, Suzhou Cancer Center, Suzhou Hospital Affiliated to Nanjing Medical University Suzhou, Jiangsu, China	Phase 1 Phase 2
NCT03940833	unpublished	Biological: BCMA CAR-NK 92 cells	Multiple Myeloma	Recruiting	Department of Hematology, Wuxi People’s Hospital, Nanjing Medical University Wuxi, Jiangsu, China	Phase 1 Phase 2
NCT04847466	unpublished	Drug: N-803 Drug: Pembrolizumab Biological: PD-L1 t-haNK	Gastroesophageal Junction (GEJ) Cancers Advanced HNSCC	Recruiting	National Institutes of Health Clinical Center Bethesda, MD, USA	Phase 2
NCT03824964	unpublished	Biological: Anti-CD19/CD22 CAR NK Cells	Refractory B-Cell Lymphoma	Unknown		Early Phase 1
NCT05020678	peripheral blood	Biological: NKX019	Lymphoma, Non-Hodgkin B-cell Acute Lymphoblastic Leukemia Large B-cell Lymphoma (and 7 more)	Recruiting	Colorado Blood Cancer InstituteDenver, CO, USA University of Chicago Chicago, IL, USA The Cleveland Clinic Foundation Cleveland, OH, USA (and 4 more…)	Phase 1
NCT02944162	NK-92 cell line	Biological: anti-CD33 CAR-NK cells	Acute Myelogenous Leukemia Acute Myeloid Leukemia Acute Myeloid Leukemia With Maturation (and 2 more)	Unknown	PersonGen BioTherapeutics (Suzhou) Co., Ltd. Suzhou, Jiangsu, China	Phase 1 Phase 2
NCT03579927	umbilical Cord Blood	Procedure: Autologous Hematopoietic Stem Cell Transplantation Drug: Carmustine Drug: Cytarabine (and 5 more…)	CD19 Positive Mantle Cell Lymphoma Recurrent Diffuse Large B-Cell Lymphoma (and 4 more)	Withdrawn	M D Anderson Cancer Center Houston, TX, USA	Phase 1 Phase 2
NCT05182073	peripheral blood	Drug: FT576 Drug: Cyclophosphamide Drug: Fludarabine Drug: Daratumumab	Multiple Myeloma Myeloma	Recruiting	Colorado Blood Cancer Institute Denver, CO, USA Tennessee Oncology—Nashville Nashville, TN, USA	Phase 1
NCT05248048	unpublished	Biological: CAR-T infusion	Refractory Metastatic Colorectal Cancer	Recruiting	The Third Affiliated Hospital of Guangzhou Medical University Guangzhou, Guangdong, China	Early Phase 1
NCT05410717	peripheral blood	Biological: Claudin6 targeting CAR-NK cell	Stage IV Ovarian Cancer Testis Cancer, Refractory Endometrial Cancer Recurrent CAR NK	Recruiting	The Second Affiliated Hospital of Guangzhou Medical University Guangzhou, Guangdong, China Guangzhou, Guangdong, China	Phase 1 Phase 2
NCT05247957	unpublished	Biological: CAR-NK cells	Safety and Efficacy	Recruiting	Hebei Yanda Lu Daopei HospitalSanhe, Hebei, China	Phase 1
NCT05472558	cord blood	Biological: anti-CD19 CAR-NK	B-cell Non Hodgkin Lymphoma	Not yet recruiting	2nd Affiliated Hospital, School of Medicine, Zhejiang University Hanzhou, Zhejiang, China	Phase 1
NCT04623944	unpublished	Biological: NKX101—CAR NK cell therapy	Relapsed/Refractory AML AML, AdultMDS Refractory Myelodysplastic Syndromes	Recruiting	Colorado Blood Cancer Institute Denver, CO, USA Winship Cancer Institute, Emory University Atlanta, GA, USA University of Chicago Medical Center Chicago, IL, USA (and 4 more)	Phase 1
NCT05410041	unpublished	Biological: CAR-NK-CD19 Cells	Acute Lymphocytic Leukemia Chronic Lymphocytic Leukemia Non Hodgkin Lymphoma	Recruiting	Beijing Boren Hospital Beijing, Beijing, China	Phase 1
NCT05336409	unpublished	Biological: CNTY-101 Biological: IL-2 Drug: Lymphodepleting Chemotherapy	R/R CD19-Positive B-Cell Malignancies Indolent Non-Hodgkin LymphomaAggressive Non-Hodgkin Lymphoma	Not yet recruiting		Phase 1

## Supplementary Materials

The following supporting information can be downloaded at: https://www.mdpi.com/article/10.3390/cancers14174318/s1, Table S1: Summary of differentiation and expansion protocols for NK cells.

## Abbreviations

aa	amino acid;
aAPC	artificial antigen-presenting cell;
AAV	adeno-associated virus
ACT	adoptive cell therapy;
ADCC	antibody-dependent cell-mediated cytotoxicity;
AICD	activation-induced cell death;
ALL	acute lymphoblastic leukemia;
BBB	blood-brain barrier;
CAR	chimeric antigen receptor;
CAR-NK	CAR-modified NK cells;
CHL	classical Hodgkin’s lymphoma;
CLL	chronic lymphocytic leukemia;
CRS	cytokine release syndrome;
CTL	cytotoxic T lymphocytes;
DLBCL	diffuse large B-cell lymphoma;
DLL1	Delta-like-1;
EBs	embryoid bodies;
EGFR	epidermal growth factor receptor;
ES	embryonic stem cells;
FasL	Fas ligand;
GM-CSF	granulocyte macrophage colony stimulating factor;
GMP	good manufacturing practice;
GvHD	graft-versus-host disease;
HLA-I	human leukocyte antigen-I;
HSC	hematopoietic stem cells;
ICANS	immune effector cell-associated neurotoxicity syndrome;
IFN-γ	interferon-γ;
IL	interleukin;
iPSCs	induced pluripotent stem cells;
ITAM	immunoreceptor tyrosine-based activation motif;
KIRs	killer cell immunoglobulin-like receptors;
KLRs	killer lectin-like receptors;
LILRs	leukocyte immunoglobulin-like receptors;
mbIL-21	membrane-bound IL-21;
MDSC	myeloid-derived suppresive cell;
MNC	mononuclear cell;
NHL	non-Hodgkin’s lymphoma;
PB	peripheral blood;
PBMCS	peripheral blood mononuclear cells;
PB-NKs	peripheral blood-derived NK cells;
PD-1	programmed cell death protein 1;
scFv	single-chain fragment variable;
sgp130	soluble glycoprotein130;
sIL-2Rα	soluble interleukin 2 receptor-α;
TAA	tumor-associated antigen;
TAM	tumor-associated macrophage;
TCM	central memory T cell;
TCRs	T cell receptors;
TEM	effector memory T cells;
Th1	T helper 1;
TM	transmembrane domain;
TME	tumor microenvironment;
TN	naive T cells;
TNF-α	tumor necrosis factor-α;
TRAIL	TNF-related apoptosis-inducing ligand;
TRAILR	TRAIL receptor;
Treg	regulatory T cells;
UCB	umbilical cord blood;
UCB-NKs	Umbilical cord blood-derived NK cells
